# Role of plasma EBV DNA levels in predicting recurrence of nasopharyngeal carcinoma in a western population

**DOI:** 10.1186/1471-2407-12-208

**Published:** 2012-05-30

**Authors:** Daris Ferrari, Carla Codecà, Cecilia Bertuzzi, Francesca Broggio, Francesca Crepaldi, Andrea Luciani, Irene Floriani, Mohssen Ansarin, Fausto Chiesa, Daniela Alterio, Paolo Foa

**Affiliations:** 1 Division of Medical Oncology and Department of Medicine, Surgery and Dentistry, San Paolo Hospital and University of Milan, Via Di Rudinì 8, Milan, Italy; 2 Istituto di Ricerche Farmacologiche Mario Negri, Via La Masa, Milan, Italy; 3 Head and Neck Surgery, European Institute of Oncology, Via Ripamonti, Milan, Italy; 4 Division of Radiotherapy, European Institute of Oncology, Via Ripamonti, Milan, Italy

**Keywords:** Nasopharyngeal carcinoma, Epstein-Barr virus, Chemoradiotherapy, Induction chemotherapy, Plasma EBV DNA copy numbers

## Abstract

**Background:**

Loco-regionally advanced nasopharyngeal carcinomas can be cured by the combination of chemotherapy and radiotherapy. In Eastern countries, plasma levels of viral Epstein-Barr deoxyribonucleic acid (DNA) are accurate in predicting recurrence, but few data are available in Western populations. The aim of this prospective study was to evaluate the relationship between viral Epstein-Barr DNA copy numbers in plasma and the response rate, progression-free survival and overall survival in a cohort of Western patients with stage IIb-IVb nasopharyngeal cancer.

**Methods:**

We evaluated plasma samples from 36 consecutive patients treated with induction chemotherapy followed by chemoradiation. EBV copy numbers were determined after DNA extraction using real-time quantitative polymerase chain reaction. Survival curves were estimated using the Kaplan–Meier method.

**Results:**

Circulating Epstein-Barr virus DNA levels were measured before treatment, at the end of concomitant chemo- and radiotherapy, and during the follow-up period. Pre-treatment levels significantly correlated with the initial stage and probability of relapse. Their increase was 100% specific and 71.3% sensitive in detecting loco-regional or metastatic recurrence (an overall accuracy of 94.4%). Three-year progression-free and overall survival were respectively 78.2% and 97.1%.

**Conclusions:**

The results of this study confirm that patients from a Western country affected by loco-regionally advanced nasopharyngeal carcinoma have high plasma Epstein-Barr virus DNA levels at diagnosis. The monitoring of plasma levels is sensitive and highly specific in detecting disease recurrence and metastases.

## Background

Nasopharyngeal carcinomas (NPCs) are frequent in South-east Asia, China, the Arctic regions and North Africa, with the highest prevalence being observed in China’s Guangdong province and in Sarawak [1-7]. However, they are rare in Europe and, in Italy, prevalence is about 1.4 per 100,000 inhabitants [8]. They are often diagnosed late because the symptoms are subtle in the early stage of disease. In Eastern populations, most stage I-II tumours are successfully treated by means of radiotherapy (RT), and about 50% of the patients with loco-regionally advanced disease (stage III-IV) are cured by means of combined chemotherapy and RT [9-12]. There are very few screening procedures for early disease [13], but repeated serological screening can be helpful in endemic regions [14]. A definite diagnosis depends on a combination of clinical suspicion, endoscopy with biopsy, and mainly magnetic resonance (MR) and positron emission tomography (PET) imaging techniques. The World Health Organization classifies NPCs as keratinising squamous carcinomas, which are characterized by well-differentiated keratin-producing cells (type I); non-keratinizing squamous cell carcinoma, differentiated (type IIa); non-keratinizing squamous cell carcinomas, undifferentiated (type IIb); basaloid squamous cell carcinomas (type III) [15].

Epidemiological studies have shown that the gamma–herpes Epstein-Barr virus (EBV) plays a major role in the pathogenesis of NPCs as they are endemic in areas where EBV infection is also endemic [16-18]. Potentially EBV-induced carcinogenesis depends on the expression of a set of oncogenes: in particular, latent membrane protein 1 (LMP1) plays a role in preventing apoptosis and activating a number of signalling pathways such as nuclear factor k-B (NF-kB), mitogen-activated protein (MAP) kinases, and phosphoinositol-3-kinase (PI3K), thus favouring cell motility and suppressing immunogenic responses [19,20].

Following the demonstration of EBV in histological samples of NPCs [21,22] and the presence of high titres of IgA antibody against a viral capside antigen (VCA) [23,24], it has been recently demonstrated that real-time quantitative polymerase chain reaction (PCR) can easily detect cell-free EBV DNA in the serum of NPC patients [25-29]. Previous studies have found different percentages of patients whose plasma was positive for EBV DNA and, sometimes, a significant proportion of patients with a low viral load, possibly due to differences in PCR methods [30]. The great majority of patients in endemic countries have high circulating levels of EBV DNA at diagnosis [31], and the higher the level, the greater the risk of recurrence. Furthermore recurrent/metastatic disease can be heralded by an increase in plasma EBV DNA levels during follow-up [26].

There are few data concerning the role of EBV DNA in Western populations, but an Italian study has demonstrated similar behaviour in a low-incidence region in which EBV DNA may be of prognostic value [32]. The aim of this prospective study was to evaluate EBV DNA levels before treatment and during follow-up in a cohort of Western patients with loco-regionally advanced NPCs.

## Methods

Untreated patients with pathologically confirmed stage IIb-IVb NPC [33] were considered eligible for study inclusion. The pre-treatment evaluation included a physical examination, the assessment of performance status, a complete blood cell count with differential, a biochemical profile, the quantification of plasma EBV DNA copy numbers, MR imaging, and the measurement of index lesions. The baseline imaging techniques included PET and computed tomography (CT) in the case of suspected metastasis.

The patients were treated by means of induction chemotherapy followed by concomitant chemo-radiotherapy (CRT). Induction chemotherapy consisted of three cycles of cisplatin (CDDP) 100 mg/m^2^ i.v. on day 1 and 5-fluorouracil (5-FU) 1000 g/m^2^/day administered as a continuous intravenous infusion on days 1–4 (PF regimen), or three cycles of CDDP 75 mg/m^2^ i.v. on day 1, docetaxel 75 mg/m^2^ i.v. on day 1, and 5-FU 750 mg/m^2^/day administered as a continuous intravenous infusion on days 1–4 (TPF regimen), both repeated every three weeks. The chemotherapy was administered via a central venous catheter in an outpatient setting. During the TPF regimen, prophylactic antibiotics were given between the cycles. During RT, CDDP was administered as a 100 mg/m^2^ intravenous infusion 60 minutes before radiation on days 1, 22 and 43. The patients were hydrated with mannitol and/or furosemide in order to maintain a high urine output. The intravenous anti-emetics given before CDDP included granisetron 3 mg and dexamethasone 8 mg.

Three-dimensional conformal RT was delivered in single daily fractions of 2 Gy on five days a week without interruptions. The planned total dose to the tumour and involved lymph nodes was 70 Gy in 35 fractions.

Tumour response was assessed by means of a clinical examination, endoscopy and MR using the response evaluation criteria in solid tumours (RECIST) [34] at the end of chemotherapy and 8–12 weeks after the completion of CRT. The patients were followed up by means of a clinical examination every two months during the first year, every three months for the following two years, and every six months thereafter. Salvage treatment after a relapse that was not eligible for surgery or re-irradiation consisted of four cycles of carboplatin AUC 5 and paclitaxel 175 mg/m^2^ on day 1 q 21.

### Tissue samples

Formalin-fixed paraffin-embedded (FFPE) tissue blocks were retrieved from the archives of the Pathology Unit. Three 10 μm thick sections from each block were collected in a 1.5 mL tube, dewaxed with xylene, dehydrated with ethanol, and incubated overnight in a digestion buffer containing 50 mM Tris, pH 8, 5 mM EDTA, pH 8, 0.5% Tween20, and 10 μL proteinase K (20 mg/ml). After digestion, total DNA was purified using DNA QIAamp DNA MiniKit (Qiagen, Hilden, Germany) spin columns as indicated by the manufacturer. The quality of the DNA was tested by means of the multiplex amplification of four fragments of 100, 200, 300 and 400 bp as previously described [35], and was considered acceptable in the presence of at least the 100, 200 and 300 bp ladders. For each sample, 5 μL of total DNA was tested for the presence of the EBV DNA IR3 region (EBNA1) using previously described primers [36]. The EBNA 1 PCR is a simple method to detect EBV DNA even if in situ hybridization for EBV-encoded RNA (EBER-RISH) can be considered the gold standard. The PCR was performed in a mixture containing 10 pmol of each primer (forward: 5’-GACGAGGGGCCAGGTACAGG-3’; reverse: 5’-GCAGCCAATGCTTCTTGGACGTTTTTGG-3’), 0.2 mmol/L dNTPs, 1.5 mmol/L MgCl2, and 1.25 U TaqGold polymerase (Applied Biosystems, Foster City, California, US) in a total volume of 50 μL. The thermocycling conditions consisted of 40 cycles at 94 °C for 10 min, 94 °C for 1 min, 55 °C for 1 min, and 72 °C for 1 min, followed by a final extension phase at 72 °C for 7 min (Thermal cycler ABI 9700, Applied Biosystems, Foster City, California, US). Appropriate positive and negative controls were used in each experiment. Ten microlitres of PCR products were run on a QIAxcel System (Qiagen, Hilden, Germany) and visualised using QIAxcel BioCalculator Sofware. The expected size of the PCR product was 241 bp.

### Plasma samples

EDTA plasma samples were collected, centrifuged at 1000 x g for 15 minutes, and then stored at −80 °C until further processing. EBV DNA copy numbers were evaluated before treatment, 1–2 weeks after CRT, and every six months during the follow-up period or in the case of a suspected or documented recurrence. The plasma samples were thawed and centrifuged at 20,000 x g for five minutes, and plasma DNA was extracted using a QIAamp DNA Blood MiniKit (Qiagen, Hilden, Germany). About 200–400 μL of each sample per column was used for DNA extraction, and the exact amount of extracted plasma was documented fin order to calculate the target DNA concentration. Fifty microlitres of distilled water was used to elute the DNA from the extraction column. The plasma concentrations of EBV DNA were measured by means of a real-time quantitative PCR assay of the *Bam* HI-W region of the EBV genome. The sequences of the forward and reverse primers were respectively 5'-CCCAACACTCCACCACACC-3' and 5'-TCTTAGGAGCTGTCCGAGGG-3'. A dual fluorescence-labelled oligomer, 5'-(FAM)CA CACACTACACACACCCACCCGTCTC(TAMRA)-3', was used as a probe. The real-time quantitative PCR assay and reaction set-up procedures have been previously described in detail. The *Bam* HI-W region showed a strong correlation with the *EBNA-1* PCR [28]. Real-time quantitative PCR was performed using a Rotor Gene Q (Qiagen, Hilden, Germany) analyser, and the plasma concentrations of EBV DNA were expressed as the number of copies of the EBV genome per millilitre of plasma. Values of <350 copies/mL were considered normal on the basis of the analytical sensitivity of the test. Plasma EBV DNA levels were also evaluated in 20 control samples taken from patients with head-and-neck cancers other than NPC, none of which had values of >350 copies/ml.

Informed consent was obtained from all of the patients, and the study protocol was approved by our local Ethics Committee.

### Statistical analysis

The aim of the study was to explore the relationship between plasma EBV DNA concentrations and progression-free survival (PFS). Response to treatment and overall survival (OS) were also assessed. PFS was calculated from the first day of treatment to the date of relapse. The patients who had not experienced a recurrence by the analysis were censored at their last disease assessment. OS was defined as the time from the first day of treatment to the date of death due to any cause. At the time of analysis, the patients who were not reported as having died were censored at the date they were last known to be alive. Induction chemotherapy was started within three days of the date of diagnosis in all patients. Survival curves were estimated using the Kaplan-Meier method. Plasma EBV DNA concentrations were analysed as both continuous and categorical variables (<350 or >350 copies), and compared using the Mann–Whitney rank-sum test. Given the exploratory nature of the study, no formal sample size calculation was made. All of the statistical tests were two-sided, and a p-value <0.05 was considered statistically significant. The analyses were carried out using SAS software, version 9.1 (SAS Institute, Cary, North Carolina, US).

## Results

Thirty-six consecutive Italian patients with pathologically confirmed stage IIb-IVb NPC referred to the Oncology Department of San Paolo Hospital, Milan (Italy), were enrolled between October 2005 and August 2009. According to the American Joint Committee on Cancer (AJCC) classification, 7^th^ edition (33), 11 patients (30.6%) were in stage IIb, 16 (44.4%) in stage III, six (16.7%) in stage IVa, and three (8.3%) in stage IVb. Histology was type IIa in two cases (5.6%), and type IIb in the remaining 34 (94.4%). Twenty-six patients (72.2%) were males, and the median age was 52 years (range 29–58). Table 1 shows the characteristics of the enrolled patients.

**Table 1:** Patients' characteristics

**Number of patients**	**36**
Median age (years)	52.0
Range	29-58
Sex – n (%)	
Male	26 (72.2)
WHO histology – n (%)	
IIa	2 (5.6)
IIb	34 (94.4)
AJCC stage - n (%)	
IIb	11 (30.6)
III	16 (44.4)
IVa	6 (16.7)
IVb	3 (8.3)
WHO Performance status	
0	33 (91.7)
1	3 (8.3)
Positive EBV-DNA	36 (100)

WHO = World Health Organization.

AJCC = American Joint Committee on Cancer.

EBV = Epstein-Barr Virus.

Before treatment, tumoral tissue from all of the patients contained EBV DNA. All but one patient had a plasma EBV DNA concentration of >350 copies/mL, with a median value of 4,701 (range 349–270,000) and an interquartile range (IQR) of 1,462-9,625. The stage IIb patients had significantly lower EBV DNA copy numbers (p = 0.007) than those in stages III-IV patients (median 2,420; IQR 820–3,939 *vs* median 7,520 and IQR 3187–12,745).

Thirty-one patients (86.1%) received the PF induction chemotherapy regimen and five the TPF regimen. At the end of CRT, a complete response (CR) was achieved by 30 patients (83.3%, 95% CI 67.2- 93.6%) and a partial response (PR) by the remaining six. EBV DNA levels had returned to below the threshold limit in all of the complete responders and four of the partial responders; the two partial responders with still high EBV DNA levels underwent a biopsy that failed to reveal the presence of tumour cells. No further treatment was given to the patients with a PR.

**Figure 1:**
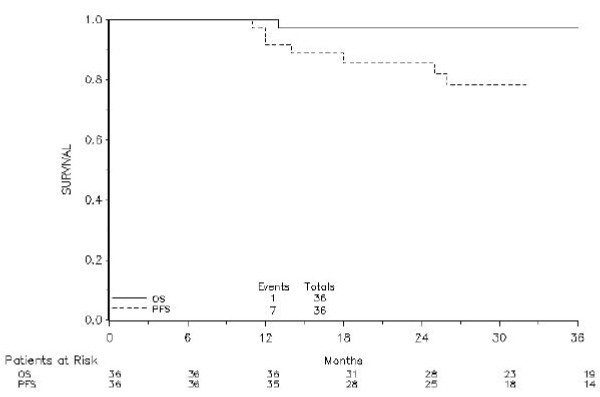
Kaplan-Meyer curves depicting progression-free survival and overall survival.

After a median follow-up of 36 months (IQR 24–48 months), seven patients had experienced recurrent disease (4 loco-regional and 3 metastatic), one of whom had died. Three-year PFS and 3-year OS were respectively 78.2% and 97.1% Figure 1.

EBV DNA load monitoring seemed to be a reliable molecular marker for predicting recurrence as five of the seven patients who relapsed (sensitivity 71.3%, 95% CI 29.0%-96.3%) had EBV DNA copy numbers that were much higher than the limit of detection (900 and 2,135 copies/mL in two patients with loco-regional relapse; and 9,185, 15,290 and 4,398,000 copies/mL respectively in three patients with metastases). The loco-regional relapse in the remaining two patients was not heralded by an increase in EBV DNA levels (Table 2) but by appearance of latero-cervical lymphadenopathy confirmed by node biopsy in one case (12 mm nodal relapse) and both nodal and nasopharyngeal biopsy in the second (respectively 11 mm and 17 mm; loco-regional relapse). MR imaging confirmed the diagnoses. The patient with the highest EBV DNA load died of disseminated disease 13 months after diagnosis.

**Table 2:** Recurring patients

**Patient No.**	**Initial stage**	**EBV DNA at diagnosis (copies/ml)**	**EBV DNA at the end of treatment (copies/ml)**	**EBV DNA at recurrence (copies/ml)**	**Time to recurrence (months)**
1	T2N3	290,000	< 350	2,135	14
2	T4N2	9,600	< 350	900	25
3	T3N2	12,745	< 350	< 350	18
4	T2N1	3,300	< 350	< 350	26
5	T2N3	87,425	7,820	4,398,000	11
6	T3N2	1,240	< 350	9,185	12
7	T2N2	7,290	1,560	15,290	12

EBV = Epstein-Barr Virus.

DNA = Deoxyribonucleic acid.

None of the 29 patients who did not experience a recurrence had EBV DNA levels above the cut-off value during the follow-up, which suggests a specificity of 100% (95%CI 88.1-100%). The overall accuracy of plasma EBV DNA levels in truly detecting recurrence was 94.4% (34/36, 95%CI 81.3-99.3).

Median plasma EBV DNA concentrations were 270 copies/mL in the patients without recurrent disease and 2,135 copies/mL in the seven experiencing recurrence (p = 0.037). Pre-treatment EBV DNA concentrations significantly correlated with PFS at univariate analysis (p = 0.036), but not after adjusting for stage (p = 0.051).

## Discussion

It has been demonstrated that NPCs are associated with EBV DNA infection as the virus infects the epithelial cells promoting the activation of proliferation signalling [37]. Tumour cells release EBV DNA during treatment, and plasma EBV DNA levels correlate with tumour volume and TNM stage [27,38-40]. Quantitative real-time PCR can detect circulating EBV DNA, and the concentration of short cell-free EBV DNA fragments can predict recurrence and survival. It has been shown that almost all NPC patients have variable pre-treatment EBV DNA copy numbers, and that the highest values correlate with a poor prognosis [41]. In the case of a CR, pre-treatment levels become undetectable after RT or CRT, whereas some patients with a PR, stable disease or progression tend to maintain high EBV DNA levels. It has been reported that patients receiving standard CRT can be stratified into subgroups on the basis of their pre- and post-treatment EBV DNA levels. Five-year OS and 5-year relapse-free survival are significantly better in the groups with low or high pre-treatment and undetectable post-treatment levels than in those with still detectable levels at the end of treatment [41].

In our study, 97% of the patients had pre-treatment levels of >350 copies/mL that significantly correlated with tumour stage (lower values in stage IIb than stages III-IV). According to Leung *et al.*[40] high pre-treatment concentrations during early-stage NPCs are associated with the risk of distant metastases, and Lin *et al.*[38] found that pre-treatment plasma EBV DNA concentrations were lower in patients with local recurrences than in those with distant metastases (1,311 *vs* 4,253 copies/mL) even if the difference was not statistically significant (p = 0.37). They also described 10 patients (10.1%) with detectable EBV DNA levels one week after completing RT, seven of whom experienced a distant relapse, which reflects the importance of undetectable plasma EBV DNA levels in maintaining a disease-free state.

Hou *et al.*[39] found that both pre- and post-treatment EBV DNA concentrations were significantly higher in their patients with distant metastases than in those with long-term disease remission or local relapse. However, in this retrospective study, the post-treatment level was more important than the pre-treatment level in predicting metastases and survival. The same conclusion applies to the study of Chan *et al.*[42], who found that a post-treatment level of >500 copies/mL in patients treated with RT significantly correlated with the poorest outcome, suggesting that this subgroup of patients should be treated more aggressively; on the contrary, levels of <500 copies/mL were associated with a better prognosis.

During the follow-up period in endemic countries, loco-regional recurrence or metastatic disease may be heralded by an increase in plasma EBV DNA levels, which can therefore be considered a useful marker for monitoring NPC patients. In a recent study, Wang *et al.* found that 36 out of 245 NPC patients (14.7%) had abnormal plasma EBV DNA levels that accurately predicted all of the NPC recurrences, and no disease was found in five patients with clinical signs of recurrence who were negative for EBV DNA. A PET scan helped in identifying the recurring lesions with a sensitivity, specificity and visual accuracy of respectively 81.8%, 77.1%, and 79.2% [43].

In our study on a cohort of patients of Italian origin, we observed three cases of metastatic spread that correlated with very high plasma EBV DNA levels, which must be attributed to an enormous load of tumour cells releasing the virus in blood while replicating. We also found that pre-treatment EBV DNA copy numbers significantly correlate with initial stage (stage IIb *vs* stage III-IV) and PFS at univariate analysis. Furthermore, the quantification of plasma EBV DNA levels proved to be extremely specific in detecting loco-regional or distant recurrences as none of the disease-free patients showed an increase. Among the relapsing patients, all three with disseminated disease were easily detected by their high EBV DNA loads, whereas the load increased to >350 copies/mL in only two of the four patients with loco-regional recurrences, thus confirming that assaying EBV DNA is less reliable in the case of a limited volume relapse.

It has recently been shown that plasma EBV DNA clearance rates have predictive and prognostic value during the first month of palliative chemotherapy in patients with metastases [44]. However, the study involved a small number of patients (34 treated with old chemotherapeutic drugs), and the unusually high response rate (41.2% CR) and unusually long OS (median 28 months), which have never reported elsewhere [45,46], suggest there may have been a selection bias. Furthermore, the need to analyse multiple plasma samples limits the usefulness of this method in clinical practice.

In a larger cohort of patients receiving chemotherapy for metastatic disease, monitoring EBV DNA levels proved to be useful in predicting which patients would survive longer (i.e. those with low pre-treatment and undetectable post-treatment levels) [47], which is in line with our experience as some of our long-term survivors maintained low EBV DNA copy numbers years after the end of chemotherapy (data not shown).

## Conclusions

In an era of new biomolecular marker development, the measurement of plasma EBV DNA levels is as essential in the West as it is in the East. Given its high degree of accuracy, all NPC patients with loco-regionally advanced disease should be regularly monitored in order to detect any increase in plasma levels and diagnose recurrence as early as possible.

## Competing interests

The authors declare that they have no competing interests.

## Authors’ contributions

DF and PF conceived the study and participated in its design and coordination. CC, CB, FB and FC participated in designing the study. AL and IF performed the statistical analysis. AM, FC and DA participated in coordinating the study. All of the authors read and approved the final manuscript.

## Pre-publication history

The pre-publication history for this paper can be accessed here:

http://www.biomedcentral.com/1471-2407/12/208/prepub
